# Genome-wide allele-specific methylation is enriched at gene regulatory regions in a multi-generation pedigree from the Norfolk Island isolate

**DOI:** 10.1186/s13072-019-0304-7

**Published:** 2019-10-08

**Authors:** Miles C. Benton, Rodney A. Lea, Donia Macartney-Coxson, Heidi G. Sutherland, Nicole White, Daniel Kennedy, Kerry Mengersen, Larisa M. Haupt, Lyn R. Griffiths

**Affiliations:** 1 Genomics Research Centre, Institute of Health and Biomedical Innovation, School of Biomedical Sciences, Queensland University of Technology, Brisbane, QLD Australia; 2 Human Genomics, Institute of Environmental Science and Research, Wellington, New Zealand; 3 ARC Centre of Excellence for Mathematical and Statistical Frontiers, Queensland University of Technology (QUT), Brisbane, QLD Australia

## Abstract

**Background:**

Allele-specific methylation (ASM) occurs when DNA methylation patterns exhibit asymmetry among alleles. ASM occurs at imprinted loci, but its presence elsewhere across the human genome is indicative of wider importance in terms of gene regulation and disease risk. Here, we studied ASM by focusing on blood-based DNA collected from 24 subjects comprising a 3-generation pedigree from the Norfolk Island genetic isolate. We applied a genome-wide bisulphite sequencing approach with a genotype-independent ASM calling method to map ASM across the genome. Regions of ASM were then tested for enrichment at gene regulatory regions using Genomic Association Test (GAT) tool.

**Results:**

In total, we identified 1.12 M CpGs of which 147,170 (13%) exhibited ASM (*P* ≤ 0.05). When including contiguous ASM signal spanning ≥ 2 CpGs, this condensed to 12,761 ASM regions (AMRs). These AMRs tagged 79% of known imprinting regions and most (98.1%) co-localised with known single nucleotide variants. Notably, miRNA and lncRNA showed a 3.3- and 1.8-fold enrichment of AMRs, respectively (*P* < 0.005). Also, the 5′ UTR and start codons each showed a 3.5-fold enrichment of AMRs (*P* < 0.005). There was also enrichment of AMRs observed at subtelomeric regions of many chromosomes. Five out of 11 large AMRs localised to the protocadherin cluster on chromosome 5.

**Conclusions:**

This study shows ASM extends far beyond genomic imprinting in humans and that gene regulatory regions are hotspots for ASM. Future studies of ASM in pedigrees should help to clarify transgenerational inheritance patterns in relation to genotype and disease phenotypes.

## Introduction

Complex human disease traits are influenced by an elaborate interplay of genetic and environmental factors [1]. Epigenetic regulation of genomic function provides a mechanism(s) allowing organisms to dynamically respond to environmental exposures [2, 3]. DNA methylation is an important epigenetic modification, which in mammals commonly occurs at cytosine-guanine dinucleotides, (referred to as CpG sites). It is well known that DNA methylation can vary among alleles at a given locus even when the DNA sequence variant is identical—termed allele-specific methylation (ASM). ASM was first recognised at imprinted loci (established in germline cells), but its presence elsewhere across the genome is indicative of its potential for more widespread importance in terms of genomic function [1]. Studies have shown that “non-imprinted” ASM is dynamic, heritable, and has been associated with non-coding genomic elements and gene expression [2, 3]. This type of ASM may allow rapid adaptation to environmental exposures and potentially play an integral role in regulatory networks involved in complex disease traits [4, 5].

With the advent of high-throughput next-generation bisulphite sequencing technologies, accurate genome-wide mapping of ASM regions is now feasible in humans. The majority of ASM work to date has been performed on inbred mouse models and human cell lines [6, 7], including the recent establishment of a resource dedicated to ASM in mice [3]. Notably, a study by Martos et al. examined genome-wide methylation patterns using a multigenerational mouse hybrid design to reveal a new paradigm of ‘switchable allele-specific DNA methylation’ [4]. Specifically, this study showed that ASM is more widespread than the known imprinted genes in mice and also can occur in both a genotype-dependent and -independent manner to influence gene expression.

In humans, the use of isolated populations, which exhibit reduced genetic and environmental diversity and contain multigenerational pedigrees, provides a logical extension to inbred mouse models for mapping ASM. The Norfolk Island (NI) population is one such genetic isolate located off the east coast of Australia. The original NI population was founded in the late 1780s on Pitcairn Island by 9 Mutineers of HMS Bounty and 6 Polynesian wives, and in 1856, the founder descendants relocated to NI. These founders have given rise to a very large pedigree containing ~ 6000 members and spanning 12 generations. Given its remoteness, this population has grown in almost complete isolation. This, along with the small island size and strict immigration policies, has meant that the genetic and environmental conditions have remained quite restricted. The NI isolate has been well characterised genetically and phenotypically as part of the Norfolk Island Health Study (NIHS) [8–10]. In this study, we applied genome-wide bisulphite sequencing to characterise ASM patterns in an NI pedigree and map ASM regions (AMRs) to known regulatory genomic features.

## Results

### Characterising the CpG landscape

Genome-wide bisulphite sequence data were obtained from peripheral blood mononucleocytes (PBMCs) for 24 individuals from a third-generation nuclear pedigree from the Norfolk Island cohort (see methods). We used the Roche Nimblegen SeqCapEpi CpGiant capture-based target enrichment protocol. This is an approach designed to capture the DNA methylation based around the sites interrogated by the Illumina 450K DNA methylation array, a well-recognised platform for genome-wide analysis of human DNA methylation. One hundred base paired-end Illumina sequencing was performed. After quality control and alignment, there was a mean coverage of 100× (range 90–160) per sample (Additional file 1: Figure S1). Mapping performance was calculated against the capture design files with all samples showing > 95% average on-target rate. The total number of CpG sites per sample ranged from 2.67 M to 7.52 M with an average of 3.48 M. When considering CpG sites, which were both on-target and had ≥ 10 times coverage, a total of 1,130,173 CpG sites were found to be present in all 24 pedigree members. Of these, 1,127,867 were autosomal CpG sites. Variability of methylation levels at these sites was observed in 168,941 (15%) CpG sites with a standard deviation > 0.1 (highly variable), using the same filter as previously described [5]. Examining the presence of single-nucleotide variants (SNVs) within a CpG site (i.e., affecting the C or G), we observed 444,330 (39.4%) CpG sites with an SNV present. Just over half (231,452) of the SNVs identified had minor allele frequency (MAF) information available. Of these, 12,670 variants had an MAF ≥ 0.05, while 26,935 showed an MAF ≥ 0.01, suggesting that the majority of the genetic variation at CpG sites was localised to ‘rare’ or as yet unassigned variants (MAF < 0.01).

### Allele-specific methylation in a three-generation pedigree

**Fig. 1:**
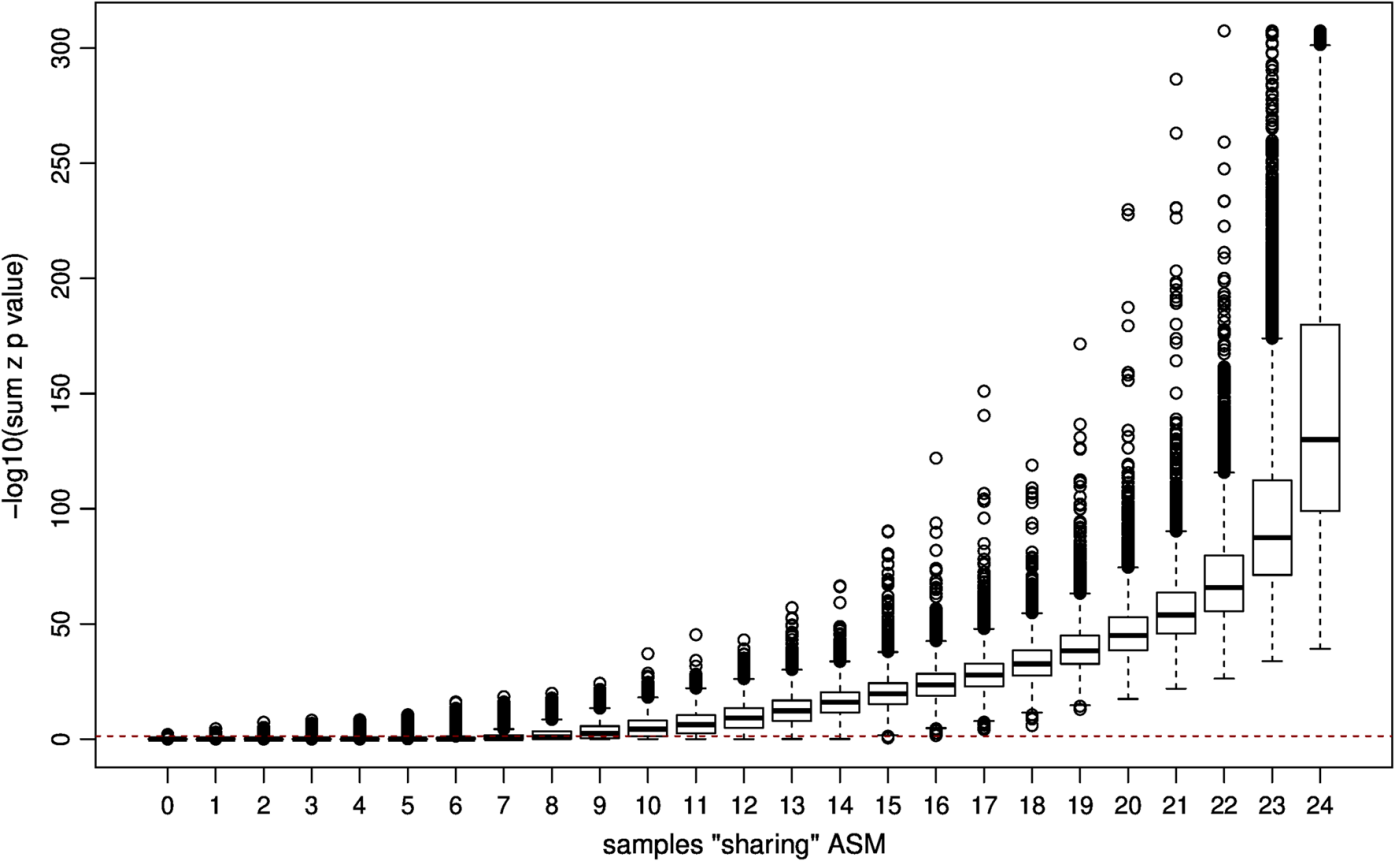
Distribution of ASM sharing among 24 members of a nuclear pedigree from the NI population

Methylation data from all subjects were analysed using a custom pipeline incorporating Methpipe software for ASM calling in a genotype-independent manner. This approach uses a probabilistic model to infer ASM by interrogating the degree to which read-based methylation states represent patterns between pairs of adjacent CpG sites [6, 7]. Adopting this approach and applying the Fisher’s Sum Z test to combine and weight individual site-specific *p* values, we identified 147,170 ASM events corresponding to a prevalence of 33% (*P* < 0.05). Approximately, 9000 ASM events were observed in all 24 pedigree members *P* < 1 × 10^−40^ (Fig. 1). Additional file 2: Figure S2 shows chromosome-specific ‘Manhattan’ plots of all ASM events and includes highlighting (dark red) of 91 known human imprinted regions, and annotation (dbSNP) of all common SNVs (MAF > 0.01, presence indicated with black asterisk).

### Identification of allele-specific methylation regions

**Fig. 2:**
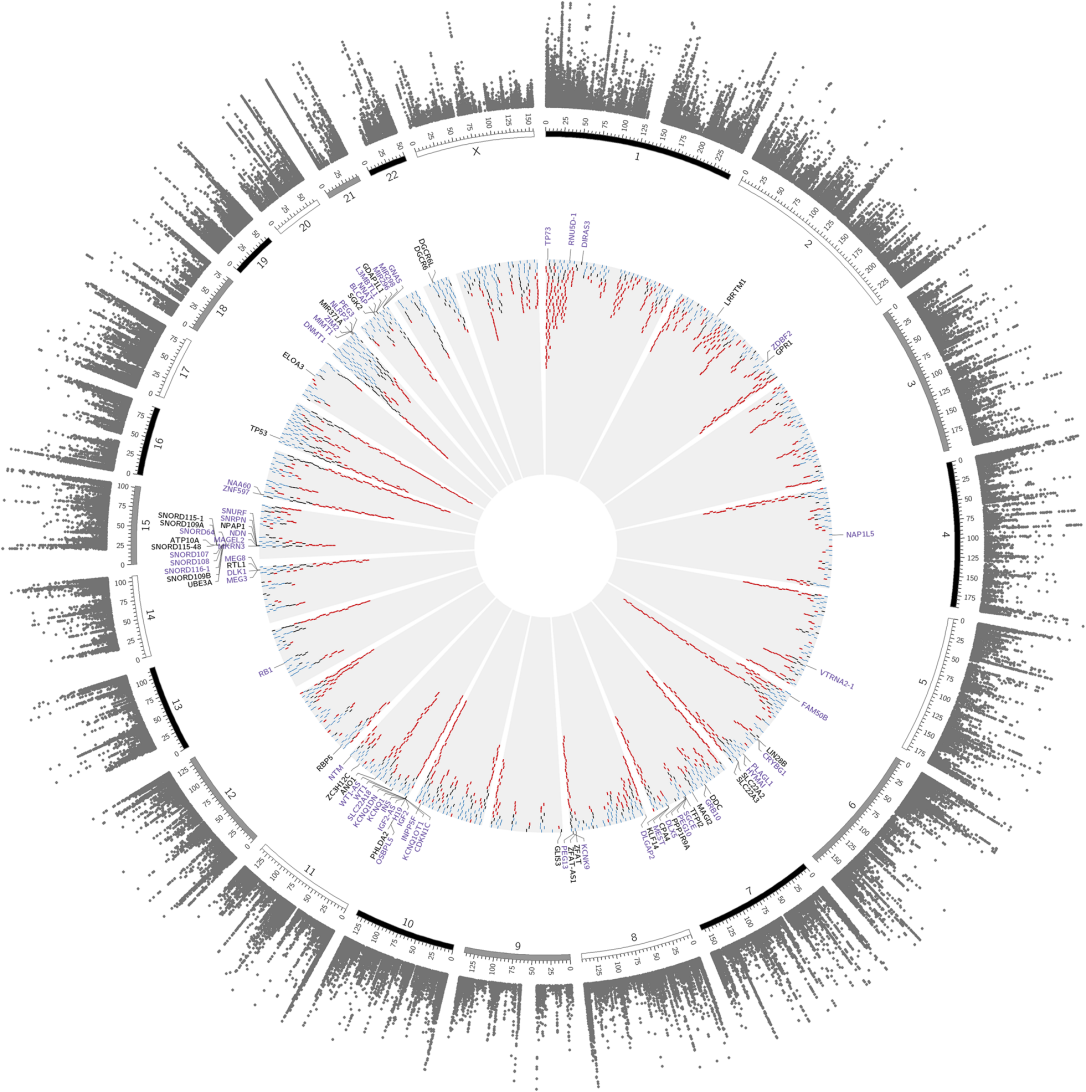
Circos plot of the genome-wide ‘landscape’ of allele-specific methylation for key Norfolk Island individuals. Circular representation of the genome, all positions indicated are relative to build hg38. The grey outermost ring represents individual CpG sites identified as exhibiting allele-specific methylation (ASM) using the Fisher’s Sum Z method −log10(*P* value). All points shown have Sum Z *P* values < 0.05, indicating ASM sharing between at least 8 individuals. The second outermost ring represents a chromosomal ideogram (annotated is the chromosomal number). The third ring annotates all 91 known human imprinted genes and their genomic position. Imprinted gene symbols indicated in purple represent imprinted genes which overlap allele-specific methylated regions (AMR). The innermost ring represents all identified AMR and their chromosomal position. A visual indication of AMR size is indicated by: blue < 1 Kb; black > 1 Kb and < 10 Kb; dark red > 10 Kb in size

An allele-specific methylation region (AMR) is defined as a genomic interval of consecutive CpG sites showing consistent ASM events. Fisher’s Sum Z test combining ASM P values across pedigree members was calculated to assign ASM sharing and a sliding window algorithm used to define ASM regions (AMRs). This approach scans the genome using a sliding window of pre-determined size to identify AMRs (consecutive ASM sites). As previously discussed and inferred by Fang et al., there is no single window size that will optimally identify AMRs through the entire genome [7]. Therefore, we adopted a similar approach to that of Fang et al. to select a window size and used a list of known human imprinted genes to test the efficacy of our sliding window criteria and the assumption that the greater the number of imprinted sites recouped the more effective the analysis. Informed by simulation analyses (see “Methods”), we defined AMRs as regions containing ≥ 2 consecutive CpG sites showing significant ASM (Fisher’s Sum Z *P* < 0.05). Using a sliding window of 5 kb, we identified 12,761 total AMRs (see innermost ring of Fig. 2 and Additional file 3: File S1).

**Fig. 3:**
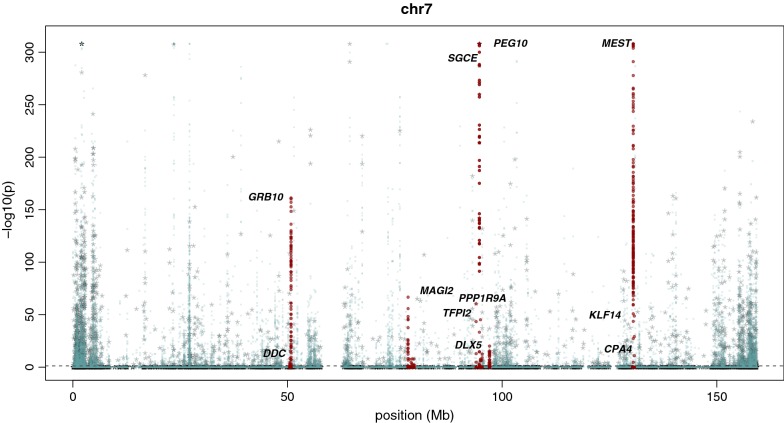
Example of ASM sharing and overlap with known imprinted genes. Sequence-based methylation mapping of 24 individuals from the NI cohort shows multiple ASM events on Chr.7 several of which map to known imprinted genes (labelled peaks) and many have co-localising SNPs (stars)

Figure 3 shows an example ‘Manhattan’ plot for chromosome 7, with confirmed imprinted loci indicated in dark-red. Interestingly, Fig. 2 illustrates obvious enrichment of AMRs at subtelomeric regions across the genome.

Parent-of-origin imprinted genes are associated with mono-allelic gene expression (accompanied by ASM of particular regions) [11]. As it is well documented that imprinted loci exhibit extensive ASM, we used a list of known human imprinted genes to validate our approach to AMR detection [12]. We obtained a curated list of human imprinted genes (http://www.geneimprint.com/) and applied several filters to remove any genes which were either predicted or conflicted as truly imprinted (see methods). This resulted in a total of 91 human imprinted genes which mapped to the genome (hg38) of which we observed an overlap of 79% (72/91) with human imprinted genes. From this analysis, the 91 known human imprinted genes and their genomic position were annotated in the third ring of Fig. 2. Imprinted gene symbols indicated in purple represent imprinted genes which overlap allele-specific methylated regions (AMR) identified in this study.

### Genomic variation and allele-specific methylation

Extending our examination of genomic variations associated with CpG sites (above), we investigated the co-localisation between common SNV (MAF ≥ 0.01 dbSNP 150) and all significant AMRs identified. To identify robust, extended AMRs, we selected regions with ≥ 10 consecutive ASM CpGs (2863/12,761, 22.4%). Using an interval-based approach with a padding of 1000 bp either side of the AMR, we identified 73 extended AMRs (73/2863, 2.5%) devoid of common SNVs, suggesting the presence of ASM events independent of genetic variation (see Additional file 4: File 2). In particular, we noted extended regions of ASM absent of common genetic variation on chromosome 21 (see Additional file 2: Figure S2).

### Enrichment of allele-specific methylated regions in potential regulatory regions

To identify ASM that overlapped gene-centric features, we next mapped the AMRs against annotated gene features (GRanges in R), which showed that the AMRs collectively overlapped with 6987 genomic features. The most commonly overlapped genomic feature was introns (34.2%), with the next highest being promoters (17.9%), followed by exons (15.1%), 1–5 kb upstream (12.8%), and intron–exon boundaries (11.3%).

**Fig. 4:**
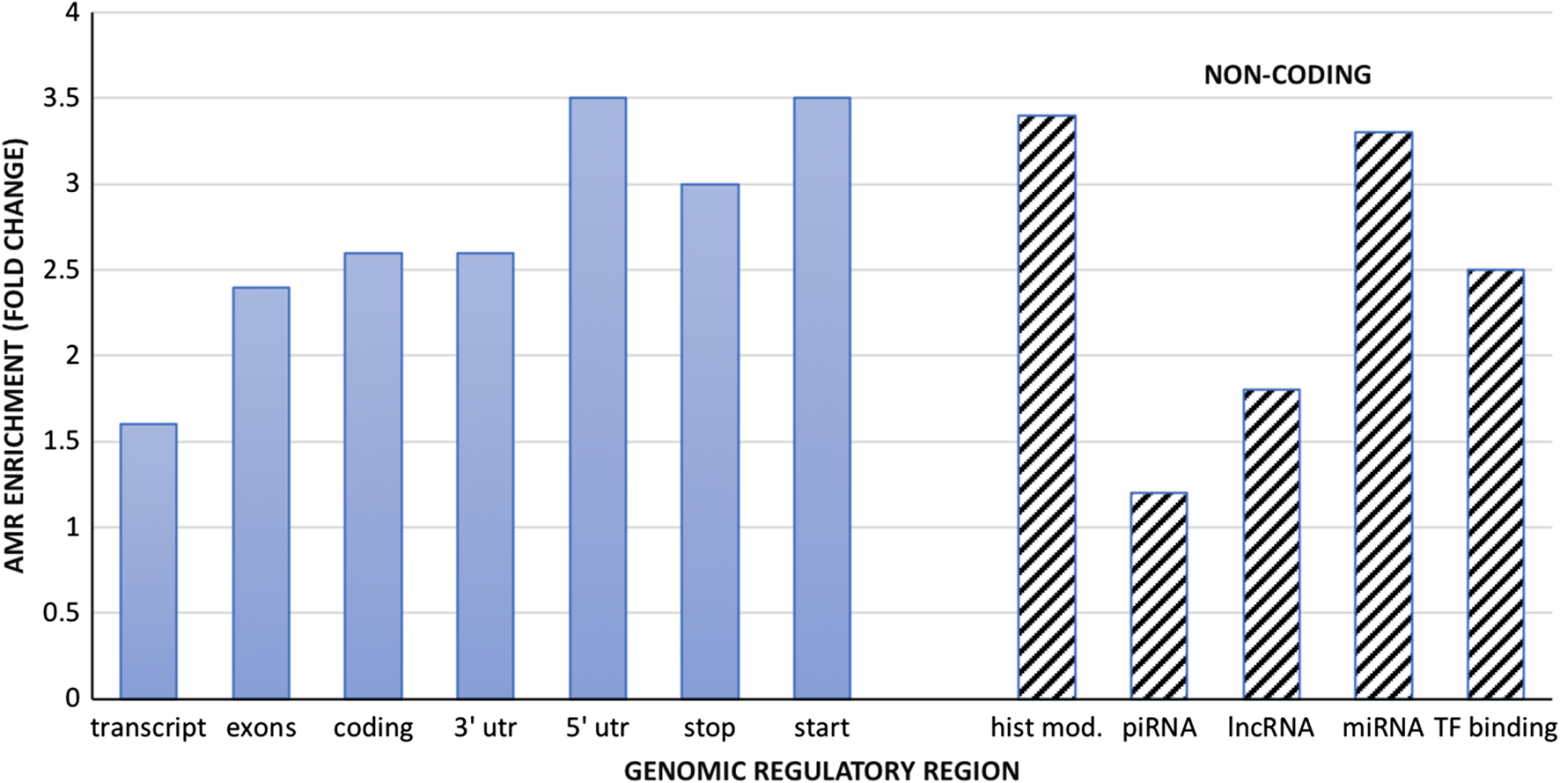
AMR enrichment at gene coding and non-coding regions in the genome

To further explore the potential functional impact of AMRs, we also investigated AMR enrichment across annotated genomic features. For this, we used Genomic Association Tester (GAT) software to perform significance testing of AMRs that overlapped functionally annotated regions and databases (i.e., GWAS catalog —https://www.ebi.ac.uk/gwas/). Figure 4 summarises significant enrichment of AMRs at regulatory genomic features. Notably, miRNA and lncRNA showed a 3.3- and 1.8-fold enrichment, respectively (*P* < 0.005), suggesting a potential importance for allele-specific regulation of these non-coding RNA. Also, the 5′ UTR and start codons each showed a 3.5-fold enrichment of AMRs (*P* < 0.005). Furthermore, there was a twofold enrichment of AMRs at GWAS loci reported in the GWAS catalogue suggesting an epigenetic contribution to complex disease traits (*P* < 0.005).

Finally, we examined large AMRs defined as containing > 200 CpGs. Of the 11 large AMRs identified, all mapped to coding regions of the genome. Interestingly, 5 out of 11 large AMRs localised to the Protocadherin cluster on chromosome 5, with another one localising to the *HOXA* cluster of developmental genes on chromosome 7 (Fig. 4 and Table 1).

**Table 1:** Localisation of large AMRs

AMR genomic position	No. CpGs	AMR width (bps)	Genes
chr1:2112321–2234147	217	121,827	*PRKCZ;SKI;FAAP20*
chr1:16624194–16728492	362	104,299	*FAM231A;MIR3675;RNVU1*-*18*
chr5:140786373–140843584	347	57,212	*PCDHA2;PCDHA3;PCDHA4;PCDHA5;PCDHA6;PCDHA7;PCDHA8;PCDHA9;PCDHA1*
chr5:140848544–140927477	263	78,934	*PCDHA10;PCDHA11;PCDHA12;PCDHA13;PCDHAC1;PCDHA9;PCDHA1;PCDHA2;PCDHA3;PCDHA4;PCDHA5;PCDHA6;PCDHA7;PCDHA8*
chr5:141121778–141208851	221	87,074	*PCDHB4;PCDHB5;PCDHB6;PCDHB7;PCDHB8;PCDHB16;PCDHB9;PCDHB10;PCDHB11;PCDHB12*
chr5:141321015–141390082	592	69,068	*PCDHGA1;PCDHGA2;PCDHGA3;PCDHGB1;PCDHGA4;PCDHGB2;PCDHGA5;PCDHGB3;PCDHGA6;PCDHGA7;PCDHGB4;PCDHGA8;TAF7*
chr5:141392580–141478530	615	85,951	*PCDHGA8;PCDHGB5;PCDHGA9;PCDHGB6;PCDHGA10;PCDHGB7;PCDHGA11;PCDHGA12;PCDHGC3;PCDHGA1;PCDHGA2;PCDHGA3;PCDHGB1;PCDHGA4;PCDHGB2;PCDHGA5;PCDHGB3;PCDHGA6;PCDHGA7;PCDHGB4*
chr6:32073900–32155177	216	81,278	*PPT2;PPT2*-*EGFL8;TNXB;ATF6B;FKBPL;PRRT1;LOC100507547*
chr7:27098296–27174773	386	76,478	*HOTAIRM1;HOXA*-*AS2;HOXA*-*AS3;HOXA10*-*AS;HOXA2;HOXA3;HOXA4;HOXA5;HOXA6;HOXA7;HOXA9;MIR196B;HOXA10;HOXA1;HOXA10*-*HOXA9*
chr21:8433975–8438909	293	4935	*LOC100861532;LOC100008587;LOC100008589*
chr21:8440273–8452215	204	11,943	*LOC100008589*

## Discussion

Studies of DNA methylation contribute to the identification of epigenetic factors that influence gene expression and susceptibility to complex disease traits. With DNA sequence variants unable to account for all of the heritability of disease, behaviors, and other multifactorial phenotypes, such studies will likely uncover information on the heritability gap left from conventional GWASs. Moreover, DNA methylation offers a natural interface for understanding the interactive effects of environmental and genetic factors (“nature” vs “nurture”) in relation to disease onset and progression.

ASM has typically been associated with genomic imprinting with studies of mouse models determining that ASM loci are far more widespread and exist with variable influence from the underlying genotype. As the patterns of ASM will vary significantly between species, studies must be performed in humans to understand disease and the impact of chemical and physical agents on their etiology [13]. Research designs that use genetically isolated human populations can be useful due to their restricted genetic and environmental variation and the presence of extended pedigrees for tracking inheritance. This potentially enriches ASM signal making it easier to detect their influence with small sample sizes.

Studying genome-wide patterns of ASM in humans has recently become more feasible with the advent of NGS bisulphite sequencing technologies. Whilst whole genome bisulphite sequencing (WGBS) remains prohibitively expensive, genome-wide bisulphite-sequencing using capture-based technology can characterise ASM for a substantial proportion of the methylome at reasonable cost. In this study, we applied this approach to a third-generation nuclear pedigree from the NI population (n = 24) and used genotype-independent methods to detect specific ASM loci and ASM regions (AMRs). Importantly, these AMRs tagged 79% of known imprinting regions, indicating the reliability of the methods. Large AMRs (> 200 CpGs and > 10 Kb in size) mapped primarily to Procadherin and *HOX* gene clusters suggesting epigenetic regulation at these transcriptionally complex regions. Protocadherin ASM has been identified in bees [14] and humans [15], and mono-allelic expression has been documented across this gene cluster previously [2, 16, 17]. In addition to the large *HOXA* AMR that we detected, ASM at some *HOXD* cluster genes (*HOXD3* and *HOXD4*) has been previously reported in blood and different regions of the brain [18]. While a large proportion of ASM appears to be conserved across tissues, tissue-specific ASM patterns have also been detected [18]. It is plausible that ASM can affect genomic control across complex regions such as Protocadherin and *HOX,* because different isoforms expressed in different cells/tissues having ASM allows for further fine tuning of these systems, potentially providing an evolutionary advantage [19].

When we tested for over-representation of AMRs with respect to annotated gene and regulatory features, we found that AMRs were highly enriched at non-coding RNA loci. At the genome-wide level, enrichment of ASM at ncRNAs and miRNAs has not been previously reported in humans, although it is known that imprinted gene domains also transcribe hundreds of miRNA, small nucleolar RNA genes (snoRNAs) [20]. Furthermore, ASM of specific non-coding genes in imprinted regions have been reported. For example, down-regulation of MEG3, a microRNA gene, by parent-specific methylation has been observed in pancreatic islets of Type 2 diabetes mellitus patients, thus providing mechanistic support for co-localisation of non-coding RNA and ASM [21].

Chromatin sites were also enriched for AMRs. Nag et al. reported a chromatin signature for monoallelically expressed genes consisting of chromatin marks associated with active transcription (H3K36me3) and silencing (H3K27me3) simultaneously occurring [22]. Similarly, we find ASM regions are enriched in histone modifications, which have been associated with both gene activity (e.g. H3K4 trimethylation, H3K9 acetylation. H3K27 acetylation, and monomethylation of H2BK5 and K4K20) and repression (e.g., trimethylation of H3K9, H3K27, and H3K79) [23]. However, the coincidence of both active H3K27 acetylation and repressive DNA methylation marks has been found at some enhancers where these bivalent regions are stabilised by, and may require DNA methylation, to potentially remain active [24].

Interestingly, there was enrichment of AMRs at subtelomeric regions, an observation not previously mentioned in other studies of AMR [15]. The subtelomeric region is important for the process of homologous chromosome recognition and pairing. The functional relevance of ASM at these genomic regions is not clear, but Law et al. showed that the X-linked gene for ATRX syndrome binds to tandem repeats at the subtelomeric regions and is involved in allele-specific expression of genes in that region [25]. It may be useful to focus larger studies on co-association of tandem repeats and ASM at sub-telomeric regions. A caveat of our observation of enrichment at sub-telomeric regions is that these chromosomal locations contain CpG dense sequences [26]. Therefore, algorithms, such as the one used here to identify ASM, which model based on adjacent CpGs may introduce a bias towards CpG dense regions; this bias would potentially affect all CpG dense regions, not just those located near the telomere. With respect to a technical bias towards enrichment of sub-telomeric regions, this may be mitigated by the capture technology which is based on gene-rich loci targeted by the Illumina 450K DNA methylation array and sub-telomeric regions are relatively gene poor [26]. It is also worth noting that DNA methylation changes within sub-telomeric regions are increasingly associated with human disease [27].

A total of 73 extended AMRs were identified to be devoid of common SNVs (MAF > 0.01) indicating non-genetic inheritance at these loci, or at least cis-acting regulation. The ASM calling approach could be considered a limitation as it is an estimation-based method. Other methods that call ASM using genomic variation (heterozygous SNVs in the reads) will likely be more accurate at directly assigning ASM, one such method was very recently published [28]. This, however, ties the calling of ASM inherently to genomic variation, whereas the method which we implemented in this study is independent of genotype, thus able to potentially identify additional regions of ASM such as that noted on chromosome 21, which was recently found to be maternally imprinted [29]. A logical step to assess the influence of genotype on methylation would be to perform mQTL analysis; however, the current sample size is too small. Tycko et al. discuss the significance of using mQTLs and ASM in combination with GWAS studies to identify disease-associated regulatory sequences [30]. They also describe various cis-acting mechanisms that lead to mQTLs and ASM, such as allele-specific histone modifications and eQTLs, from which we can learn more about the biological functions of these DNA regulatory variants, transcription factors, and pathways that interact with them.

The use of a genome-wide bisulphite sequencing approach (as opposed to WGBS) means that we could not represent all AMRs (i.e., if the AMR extends beyond the capture region, we are unable to detect this). However, to account for bias in enrichment approach, we provided the GAT software with a design file (padded 1000 bp either side of each capture region) as the search space, meaning that enrichment is restricted to the captured region (and not the whole genome). Future studies of WGBS and the use of larger extended pedigrees may provide a more accurate and comprehensive ASM map and relationship profile with regulatory regions. A study by Zink et al. has used the pedigree structure of the Icelandic population to assign parent-of-origin to transmitted alleles and methylation levels across the genome. With these data, they were able to provide new insights into imprinted regions with high-resolution maps across key regions, e.g., the Angelman syndrome/Prader-Willi locus on 15q11.2 [31].

In summary, this study has used genome-wide bisulphite sequencing to map ASM in a multi-generational pedigree from the NI genetic isolate. We have confirmed that ASM regions are widespread and extend far beyond known genomic imprinting loci in humans. Importantly, our results show that AMRs are highly enriched in non-coding genomic regions providing evidence for an integral role in gene regulatory networks. Moving research from mouse models to humans is crucial to further understand the complex interplay of allele-specific methylation and gene expression. Studies of pedigrees within genetically and environmentally limited isolates are a natural extension to mouse models. The knowledge gained from such experiments will advance our fundamental understanding of the complex patterns of epigenetic regulation in human populations, aiding in our further understanding of the epigenetic basis of complex traits and diseases.

## Methods

### DNA samples

The Norfolk Island Health Study (NIHS) was established in 2000, and several collections have since occurred as part of an extended health survey [8–10]. DNA sample collection and preparation has been previously reported [10]. Briefly, EDTA anti-coagulated venous blood samples were collected from all participants and genomic DNA was extracted from blood buffy coats using a standard salting out method. All individuals gave written informed consent. Ethical approval was granted prior to the commencement of the study by the Griffith University Human Research Ethics Committee (approval no: 1300000485) and subsequently at QUT (approval no: 1600000464). The project was performed in accordance with the relevant guidelines, which complied with the Helsinki Declaration for human research. DNA samples from 24 individuals from 3 generations of the NI core pedigree were used for this study. We chose to select individuals from multiple generations within a nuclear pedigree to capture both within- and trans-generation ASM patterns and thus provide a representative map of the NI population at large. The pedigree consisted of 13 males and 11 females with mean age of 42 years (sd = 8 years).

### Library preparation using SeqCap Epi and sequencing

DNA methylation was assayed by bisulfite sequencing using Roche SeqCap Epi CpGiant capture, which is a fixed, genome-wide targeted enrichment system, allowing interrogation of > 5.5 million methylation sites at single-nucleotide resolution. Briefly, genomic DNA was quantified on the Qubit 2.0 fluorometer using the DNA High Sense kit and 1 ug of input genomic DNA was sheared to ~ 200 bp fragments using a Covaris E220 sonicator. DNA libraries were prepared from DNA samples (including a bisulfite conversion control) according to the manufacturer’s protocol using the KAPA Biosystems HTP Library Preparation kit for Illumina platforms, which includes end repair, A-tailing, ligation to indexed 5-methyl-C modified adaptors and size selection. Bisulfite conversion of libraries was performed using Zymo Research EZ DNA Methylation-Lightning Kits and bisulfite-converted libraries were amplified via of 12 cycles of ligation mediated PCR using the KAPA HiFi HotStart Uracil + ReadyMix. Enrichment and capture of bisulfite-treated libraries were performed using the Roche Nimblegen SeqCap Epi kit, which involves hybridization to the SeqCap Epi probe pool, bead capture and further amplification of the captured bisulfite-converted DNA using the KAPA HiFi HotStart ReadyMix. Libraries were validated using an Agilent 2100 Bioanalyzer and DNA 1000 chips and reagents, and samples pooled to allow multiplex sequencing. Pre-Sequencing QC was performed on each library pool using the Bioanalyzer, and via qPCR using the KAPA Library Quant Kit for Illumina on the AB ViAA7 qPCR machine. 2 × 100 bp Paired-end sequencing of captured bisulfite-converted samples was performed on the HiSeq 4000 across three lanes. Demultiplexing was then performed to identify the separate samples on each lane, using the index reads.

### Sequence QC, processing, and methylation calling

Our processing pipeline was created to take advantage of multiple threads and parallel computing where possible. Unless otherwise stated all computing was performed on a DELL PowerEdge T620 (2 × E5-2630 6 core 24 threads, 256 GB RAM). Initial read quality assessment of fastq files was performed using fastqc (http://www.bioinformatics.babraham.ac.uk/projects/fastqc/) and adapter trimming was achieved via trimmomatic [32]. Forward and reverse reads for each sample were aligned against an in silico converted hg38 human reference genome using bowtie2 [33] as implemented in bismark, a software suite specifically designed for bisulfite sequence mapping [34]. After alignment, all multiplexed bam files for each individual were merged and sorted using sambamba (http://lomereiter.github.io/sambamba/). Deduplication of reads was conducted using the ‘MarkDuplicates’ function within Picard Tools (https://broadinstitute.github.io/picard/), ‘CollectAlignmentMetrics’ was used to compute basic alignment statistics, and ‘CalculateHsMetrics’ to calculate all hybrid capture-related metrics, including the on-target rate. Bisulfite conversion rates were calculated using the bsrate function of the methpipe package [35]. To identify and correct for methylation bias (increases/decreases in observed methylation rate at the ends of reads), the software MethylDackel (https://github.com/dpryan79/MethylDackel) was used along with a custom bash script to extract appropriate parameters from mbias plots for read correction during extraction. MethylDackel was then used to extract all CpG sites and read counts formatted for the R analysis package methylkit [36]. Single-nucleotide variant (SNV) calling from the sequence data was performed using BISCUIT (https://github.com/zwdzwd/biscuit). A list of common SNVs (MAF ≥ 0.01) was extracted from dbSNP 150. On-target rates were calculated using the SeqCapEpi CpGiant bed files provided by Roche-NimbleGen (http://sequencing.roche.com/content/dam/rochesequence/worldwide/resources/130912_HG19_CpGiant_4M_EPI.zip), bedtools intersect was used to determine on-target rates for each sample.

### Bioinformatic analysis

CpG specific QC analysis was performed in R/methylkit; all sites with < 10× coverage were discarded as well as any sites with excessive coverage (> 500×) which can be indicative of PCR bias. All overlapping CpG sites were assigned percentage methylation values and only CpG sites present in all 24 samples were retained for analysis. Allele-specific methylation (ASM) events were approximated using the software methpipe [36] and allele-specific methylated regions (AMR) were calculated using a combination of the AMR method in methpipe and a custom R script. Genomic region processing was performed in R using the package bedr (https://CRAN.R-project.org/package=bedr), genomic feature annotations were performed using the package annotatr [37]. Feature enrichment analysis was performed using Genomic Association Tester (GAT) [38]. GAT is a tool for computing the significance of overlap between multiple sets of genomic intervals. GAT estimates were significance based on simulation and can account for genome organization like isochores and correct for regions of low mapability. All enrichment analyses were run through 10,000 simulations.

## Supplementary information

**Additional file 1: Figure S1.** Coverage plot.

**Additional file 2: Figure S2.** ‘Manhattan’ plots of ASM across each Chromosome.

**Additional file 3: File S1.** Information for all 12671 AMRs.

**Additional file 4: File S2.** Information for the 73 extended AMRs without SNVs.

## Data Availability

The data that support the findings of this study are available from the Norfolk Island Government, but restrictions apply to the availability of these data, which were used under special agreement for the current study, and so are not publicly available. Data may, however, be made available from the authors upon reasonable request and with permission of the Norfolk Island Government.

## References

[1] Zhang Y, Rohde C, Reinhardt R, Voelcker-Rehage C, Jeltsch A (2009). Non-imprinted allele-specific DNA methylation on human autosomes. Genome Biol.

[2] Chess A (2005). Monoallelic expression of protocadherin genes. Nat Genet.

[3] Wasson JA, Birol O, Katz DJ (2017). A resource for the allele-specific analysis of DNA methylation at multiple genomically imprinted loci in mice. Genes Genomes Genetics.

[4] Martos SN (2017). Two approaches reveal a new paradigm of ‘switchable or genetics-influenced allele-specific DNA methylation’ with potential in human disease. Cell Discovery.

[5] Plongthongkum N, Diep DH, Zhang K (2014). Advances in the profiling of DNA modifications: cytosine methylation and beyond. Nat Rev Genet.

[6] Song Q (2013). A reference methylome database and analysis pipeline to facilitate integrative and comparative epigenomics. PLoS ONE.

[7] Fang F (2012). Genomic landscape of human allele-specific DNA methylation. Proc Natl Acad Sci.

[8] Bellis C (2005). Phenotypical characterisation of the isolated Norfolk Island population focusing on epidemiological indicators of cardiovascular disease. Hum Hered.

[9] Mackey DA (2011). The Norfolk Island Eye Study (NIES): rationale methodology and distribution of ocular biometry (biometry of the bounty). Twin Res Hum Genet.

[10] Benton MC (2013). Mapping eQTLs in the Norfolk Island genetic isolate identifies candidate genes for CVD risk traits. Am J Hum Genet.

[11] Onyango P (2002). Monoallelic expression and methylation of imprinted genes in human and mouse embryonic germ cell lineages. Proc Natl Acad Sci.

[12] Peters J (2014). The role of genomic imprinting in biology and disease: an expanding view. Nat Rev Genet.

[13] Luedi PP (2005). Genome-wide prediction of imprinted murine genes. Genome Res.

[14] Remnant EJ (2016). Parent-of-origin effects on genome-wide DNA methylation in the Cape honey bee (Apis mellifera capensis) may be confounded by allele-specific methylation. BMC Genomics.

[15] Cheung WA (2017). Functional variation in allelic methylomes underscores a strong genetic contribution and reveals novel epigenetic alterations in the human epigenome. Genome Biol.

[16] Eckersley-Maslin MA, Spector DL (2014). Random monoallelic expression: regulating gene expression one allele at a time. Trends Genet.

[17] Esumi S (2005). Monoallelic yet combinatorial expression of variable exons of the protocadherin-α gene cluster in single neurons. Nat Genet.

[18] Marzi SJ, Meaburn EL, Dempster EL, Lunnon K, Paya-Cano JL, Smith RG, Volta M, Troakes C, Schalkwyk LC, Mill J (2016). Tissue-specific patterns of allelically-skewed DNA methylation. Epigenetics..

[19] Gregg C (2014). Known unknowns for allele-specific expression and genomic imprinting effects. F1000 Prime Rep.

[20] Girardot M, Cavaillé J, Feil R (2012). Small regulatory RNAs controlled by genomic imprinting and their contribution to human disease. Epigenetics..

[21] Kameswaran V, Bramswig NC, McKenna LB, Penn M, Schug J, Hand NJ, Chen Y, Choi I, Vourekas A, Won KJ, Liu C, Vivek K, Naji A, Friedman JR, Kaestner KH (2014). Epigenetic regulation of the DLK1-MEG3 microRNA cluster in human type 2 diabetic islets. Cell Metab.

[22] Nag A, Vigneau S, Savova V, Zwemer LM, Gimelbrant AA (2015). Chromatin signature identifies monoallelic gene expression across mammalian cell types. G3 (Bethesda)..

[23] Barski A, Cuddapah S, Cui K, Roh TY, Schones DE, Wang Z, Wei G, Chepelev I, Zhao K (2007). High-resolution profiling of histone methylations in the human genome. Cell.

[24] Charlet J, Duymich CE, Lay FD, Mundbjerg K, Dalsgaard Sørensen K, Liang G, Jones PA (2016). Bivalent regions of cytosine methylation and H3K27 acetylation suggest an active role for DNA methylation at enhancers. Mol Cell.

[25] Law MJ, Lower KM, Voon HP, Hughes JR, Garrick D, Viprakasit V, Mitson M, De Gobbi M, Marra M, Morris A, Abbott A, Wilder SP, Taylor S, Santos GM, Cross J, Ayyub H, Jones S, Ragoussis J, Rhodes D, Dunham I, Higgs DR, Gibbons RJ (2010). ATR-X syndrome protein targets tandem repeats and influences allele-specific expression in a size-dependent manner. Cell.

[26] Blasco MA (2007). The epigenetic regulation of mammalian telomeres. Nat Rev Genet.

[27] Hu H, Li B, Duan S (2019). The alteration of subtelomeric DNA methylation in aging-related diseases. Front Genet..

[28] Guo W (2017). CGmapTools improves the precision of heterozygous SNV calls and supports allele-specific methylation detection and visualization in bisulfite-sequencing data. Bioinformatics.

[29] da Silva AFA (2016). Trisomy 21 alters DNA methylation in parent-of-origin-dependent and -independent manners. PLoS ONE.

[30] Zink F, Magnusdottir DN, Magnusson OT, Walker NJ, Morris TJ, Sigurdsson A, Halldorsson GH, Gudjonsson SA, Melsted P, Ingimundardottir H, Kristmundsdottir S, Alexandersson KF, Helgadottir A, Gudmundsson J, Rafnar T, Jonsdottir I, Holm H, Eyjolfsson GI, Sigurdardottir O, Olafsson I, Masson G, Gudbjartsson DF, Thorsteinsdottir U, Halldorsson BV, Stacey SN, Stefansson K (2018). Insights into imprinting from parent-of-origin phased methylomes and transcriptomes. Nat Genet.

[31] Tycko B (2010). Allele-specific DNA methylation: beyond imprinting. Hum Mol Genet.

[32] Bolger AM, Lohse M, Usadel B (2014). Trimmomatic: a flexible trimmer for Illumina sequence data. Bioinformatics.

[33] Langmead B, Salzberg SL (2012). Fast gapped-read alignment with Bowtie 2. Nat Methods.

[34] Krueger F, Andrews SR (2011). Bismark: a flexible aligner and methylation caller for Bisulfite-Seq applications. Bioinformatics.

[35] Song Q (2013). A reference methylome database and analysis pipeline to facilitate integrative and comparative epigenomics. PLoS ONE.

[36] Akalin A (2012). methylKit: a comprehensive R package for the analysis of genome-wide DNA methylation profiles. Genome Biol.

[37] Cavalcante RG, Sartor MA (2017). annotatr: genomic regions in context. Bioinformatics.

[38] Heger A, Webber C, Goodson M, Ponting CP, Lunter G (2013). GAT: a simulation framework for testing the association of genomic intervals. Bioinformatics.

