# Supplementary figures and images for: Genome-wide allele-specific methylation is enriched at gene regulatory regions in a multi-generation pedigree from the Norfolk Island isolate
Source: Epigenetics Chromatin. 2019 Oct 8;12:60. doi: 10.1186/s13072-019-0304-7 (PMC6781349; doi:10.1186/s13072-019-0304-7)

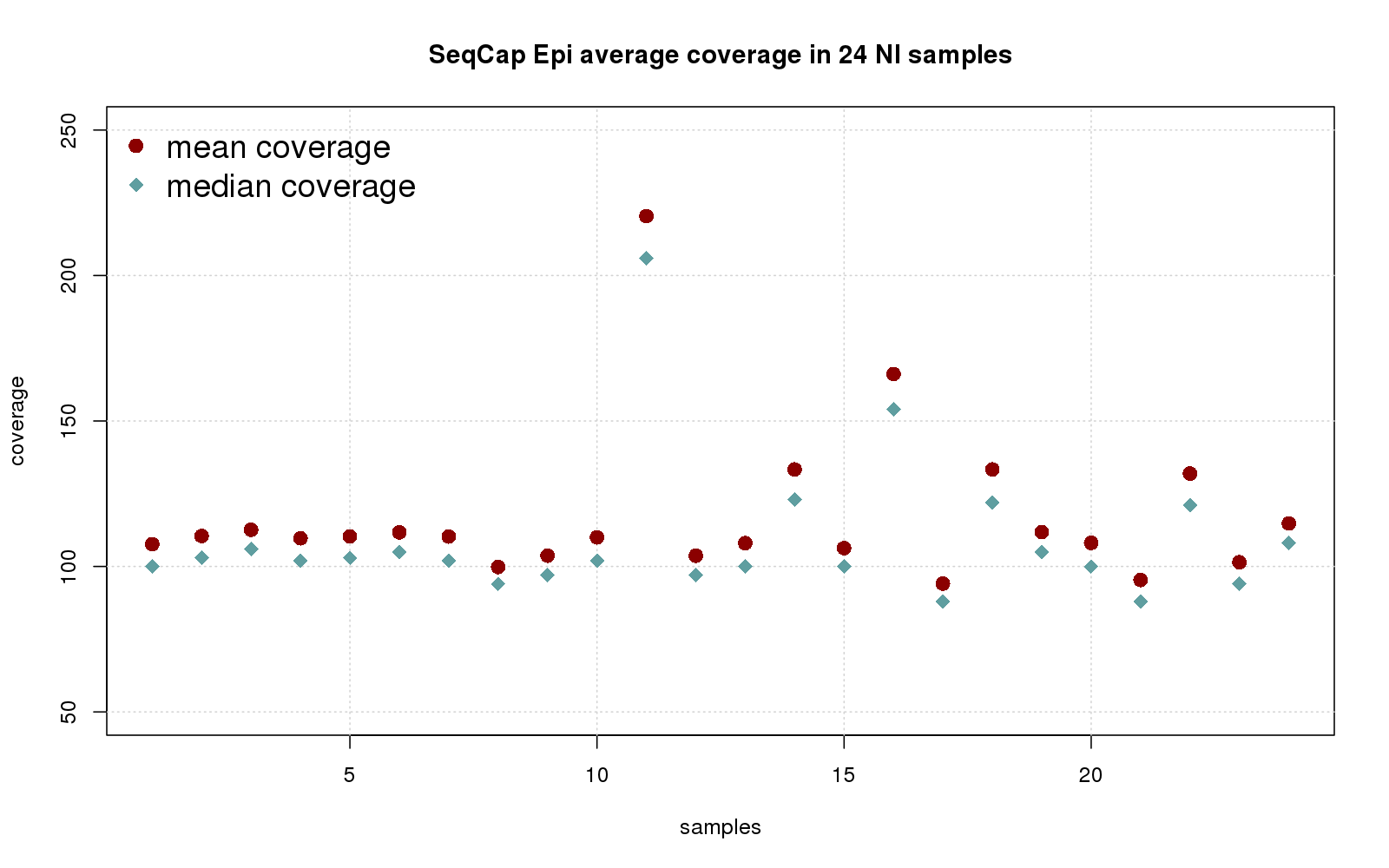

Supplement: Supplementary file 1 — Additional file 1: Figure S1. Coverage plot. [file 13072_2019_304_MOESM1_ESM.tiff]

chr1

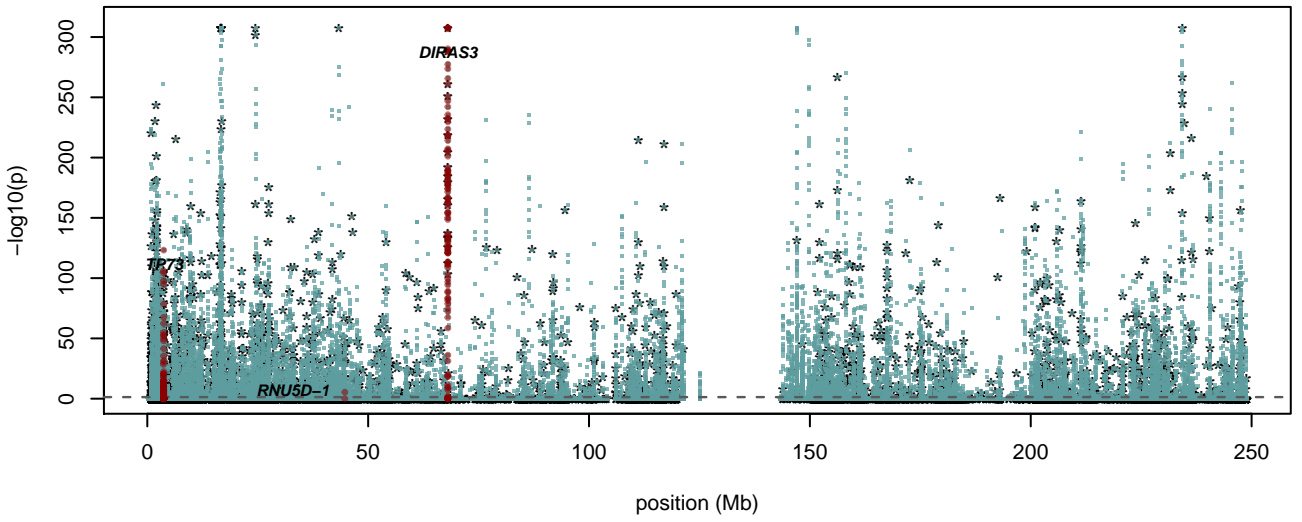

chr2

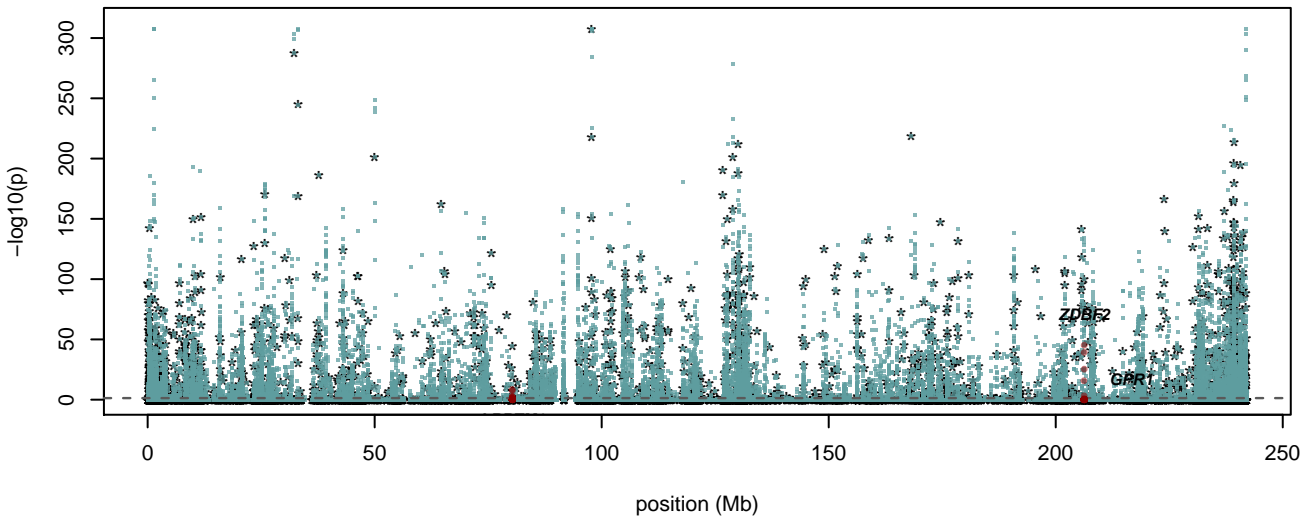

chr3

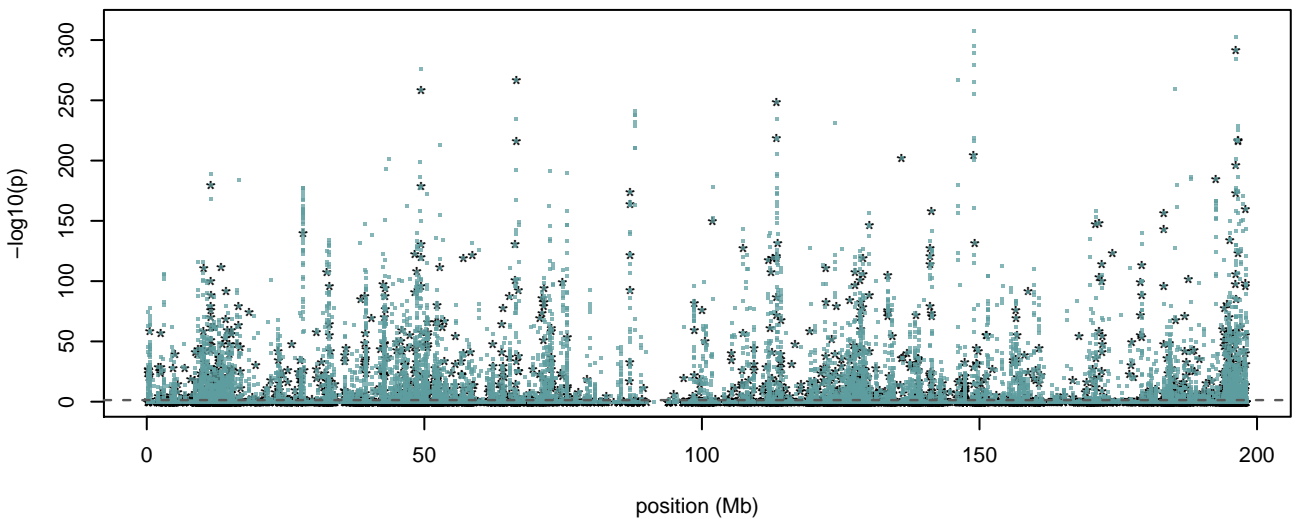

chr4

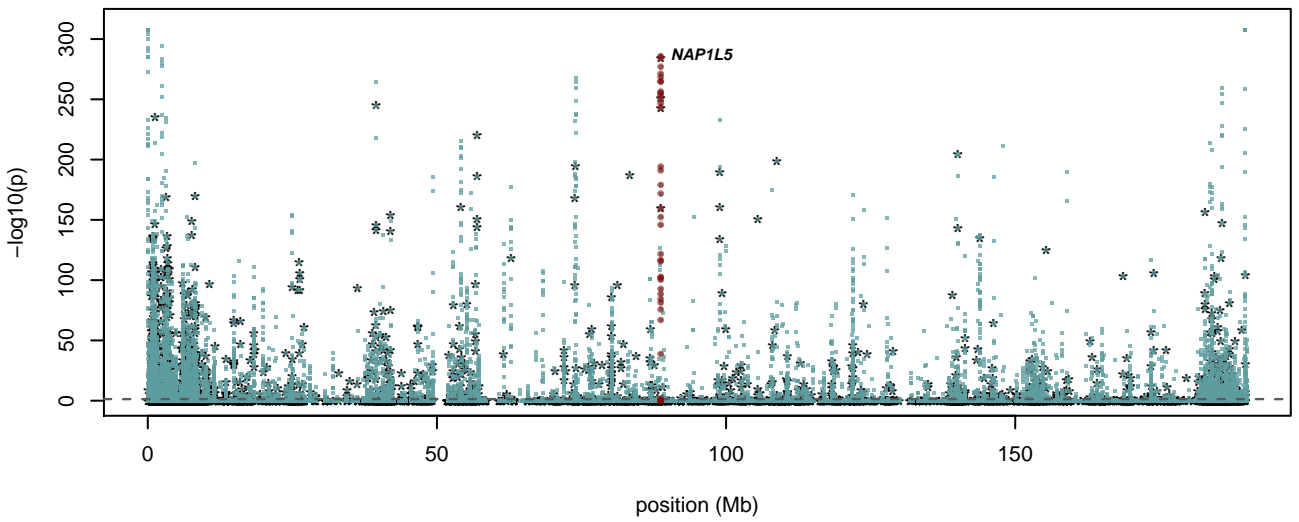

chr5

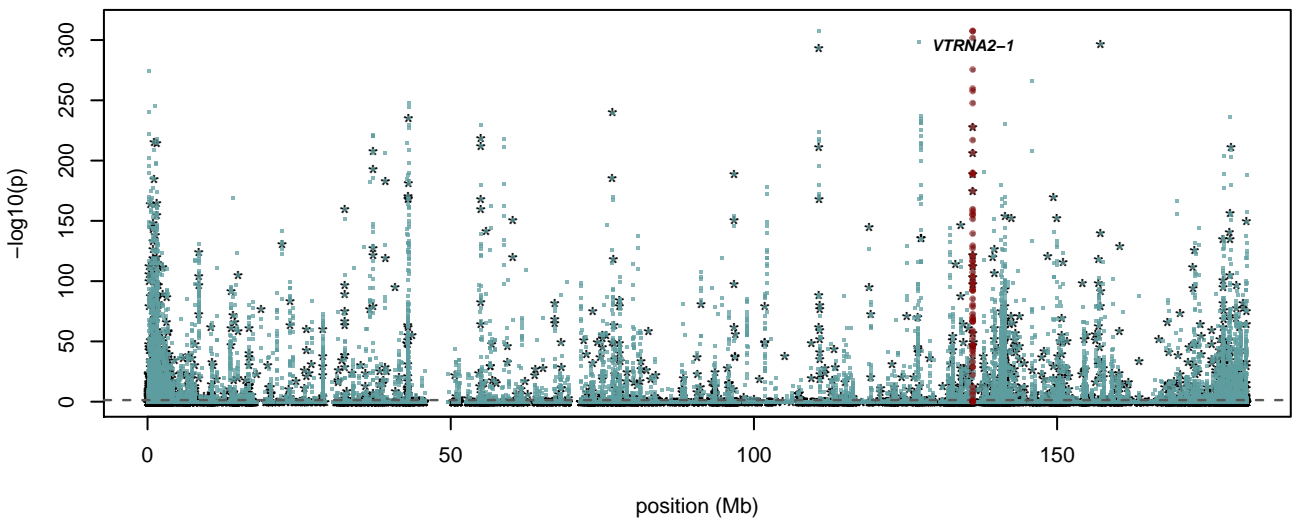

chr6

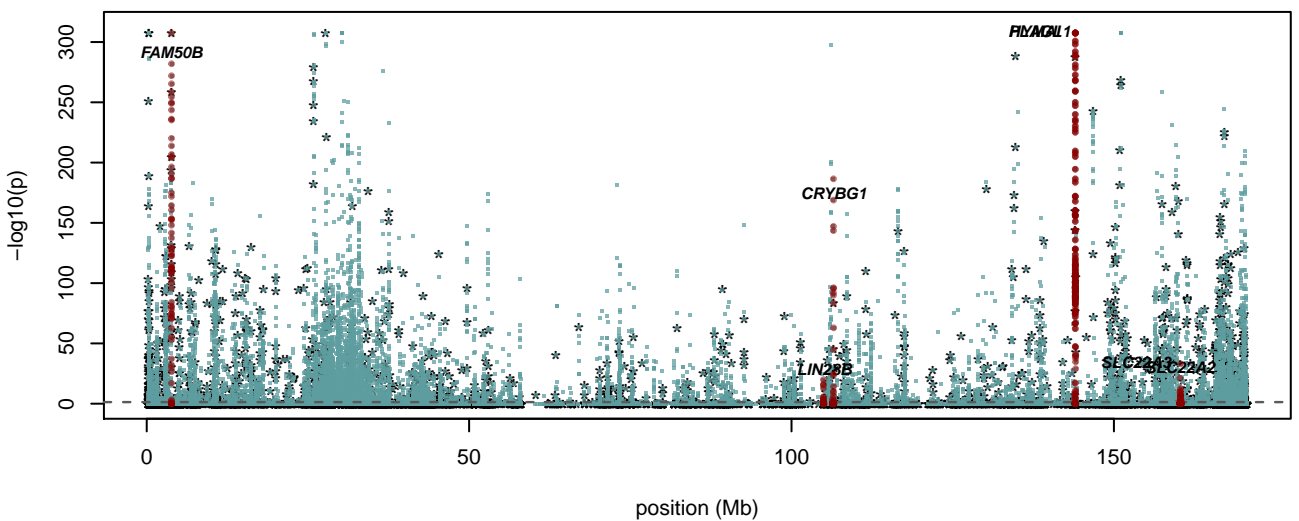

chr7

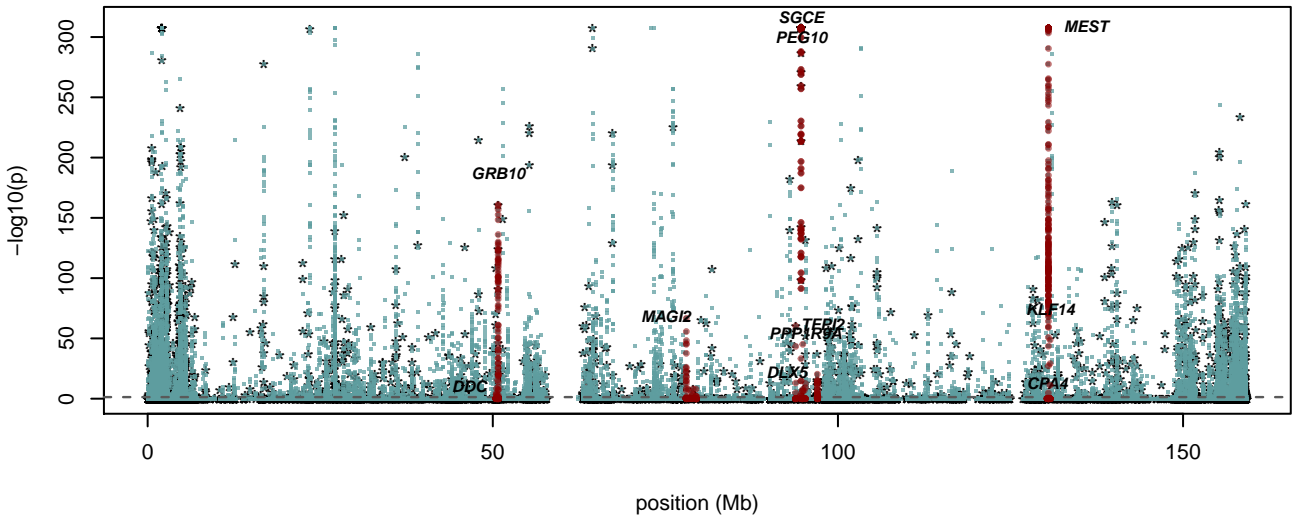

chr8

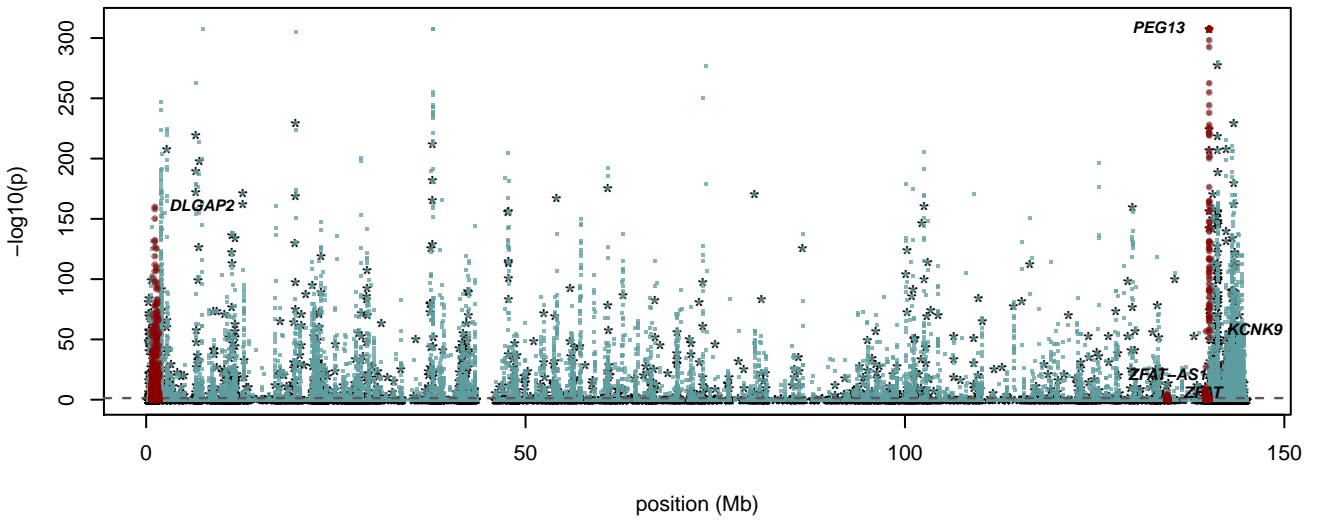

chr9

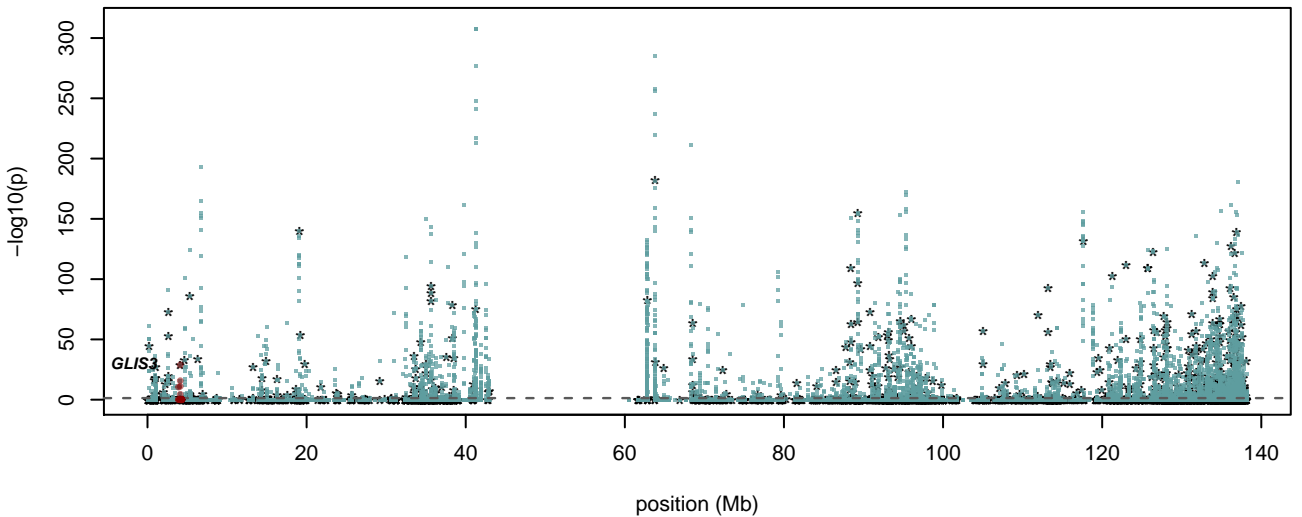

chr10

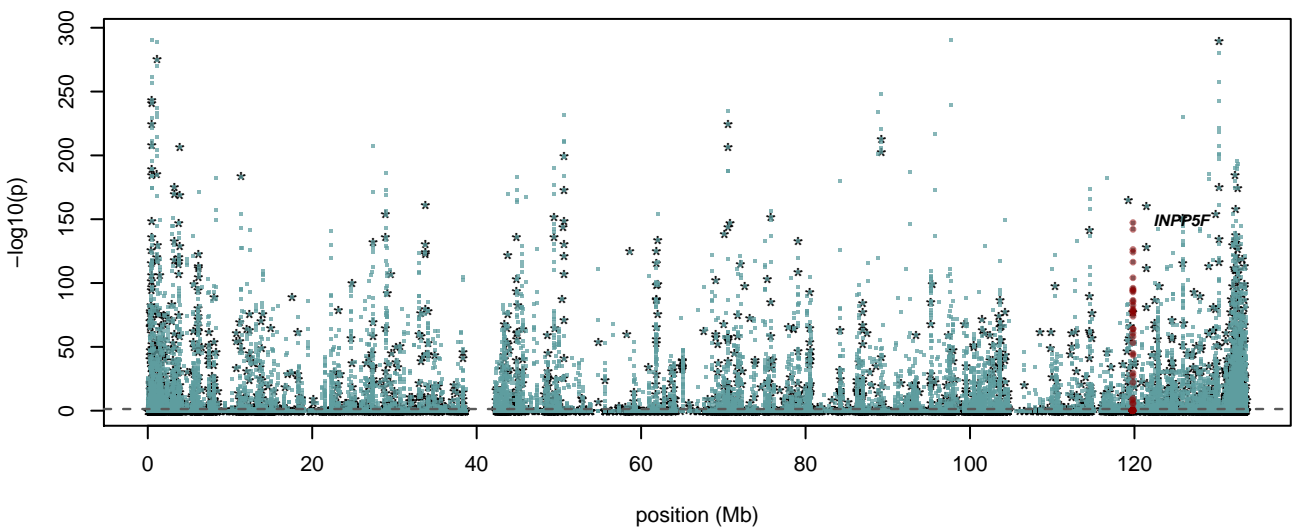

chr11

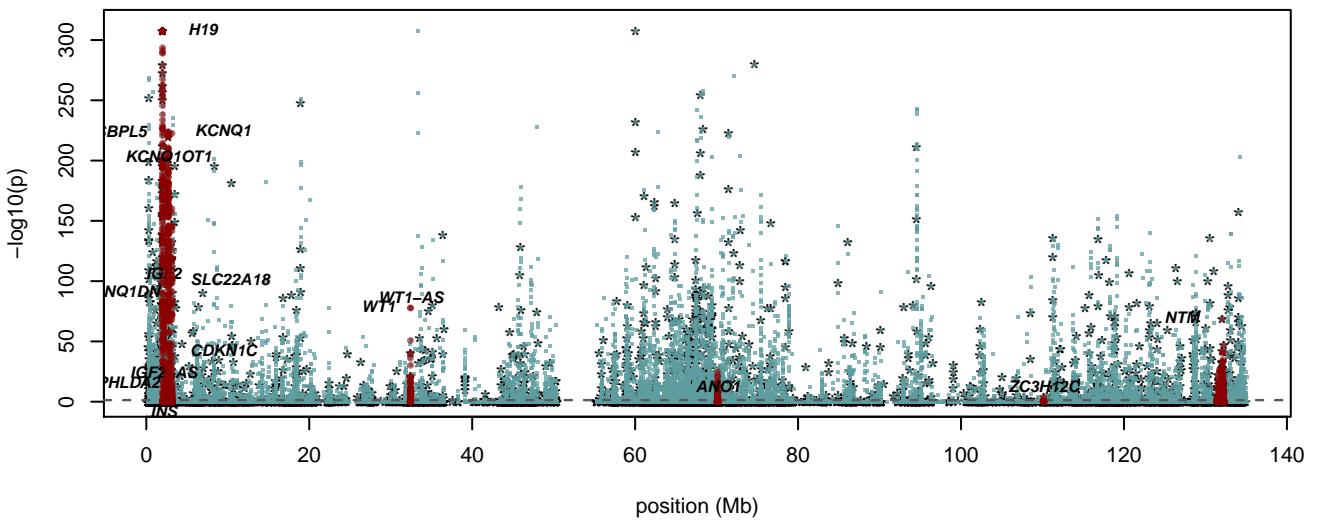

chr12

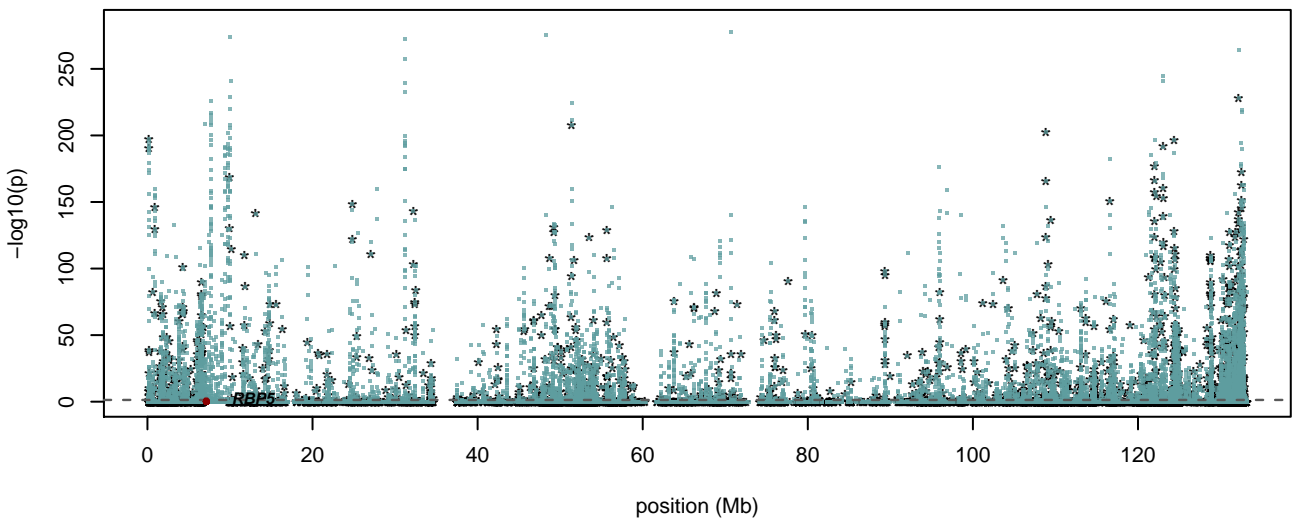



chr16

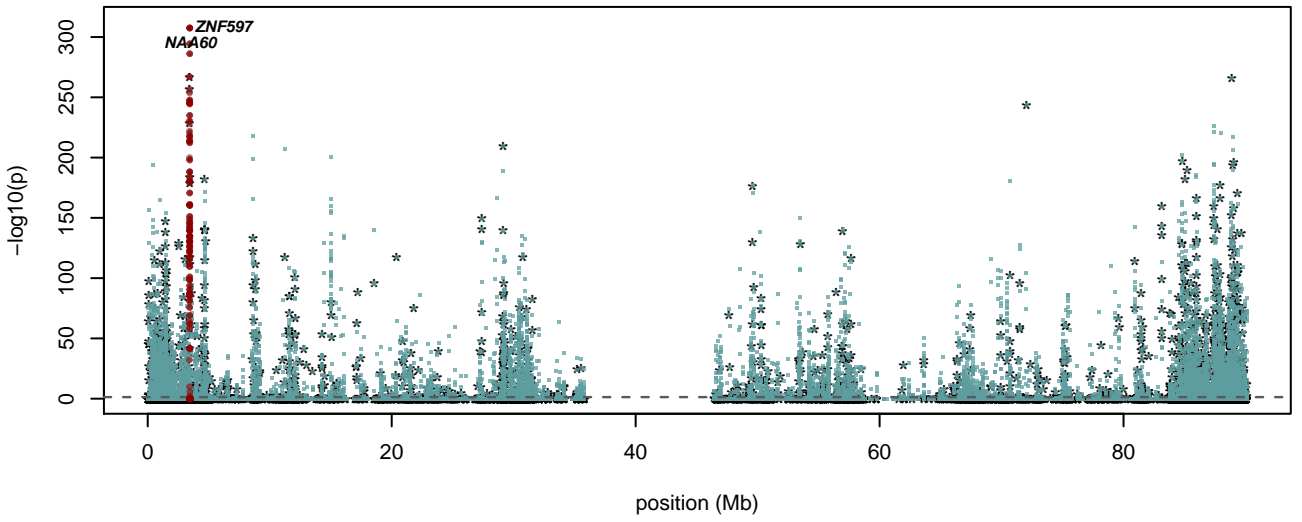

chr17

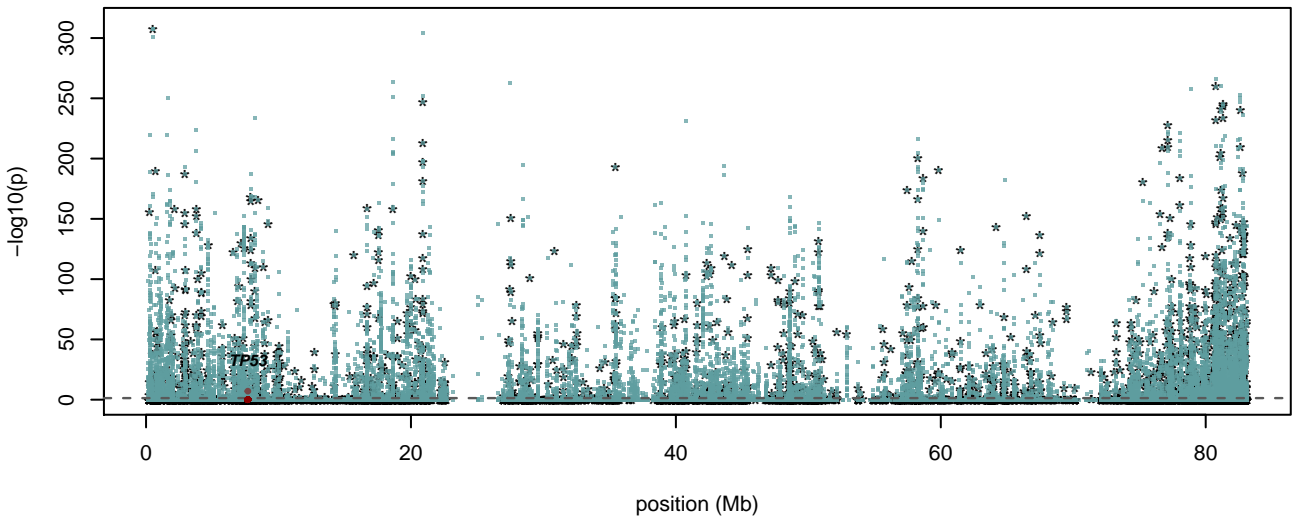

chr18

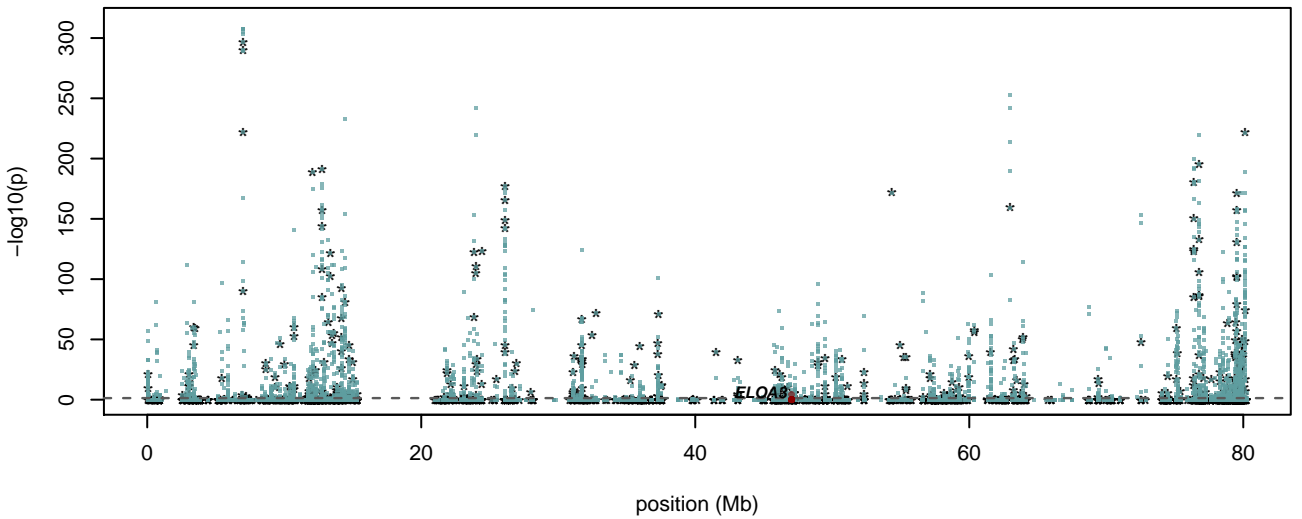

chr19

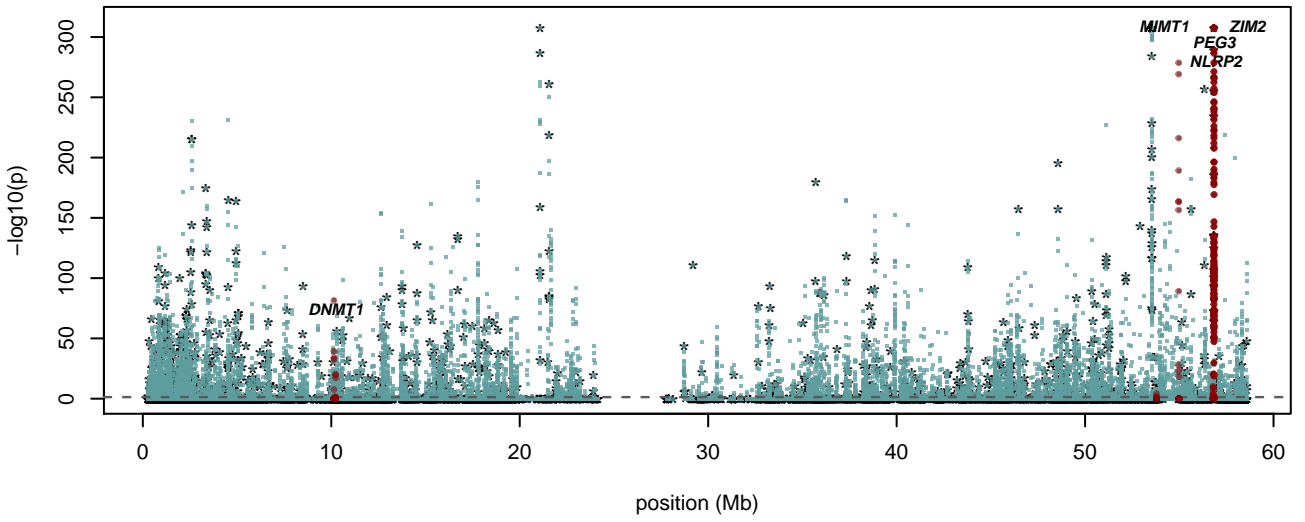

chr20

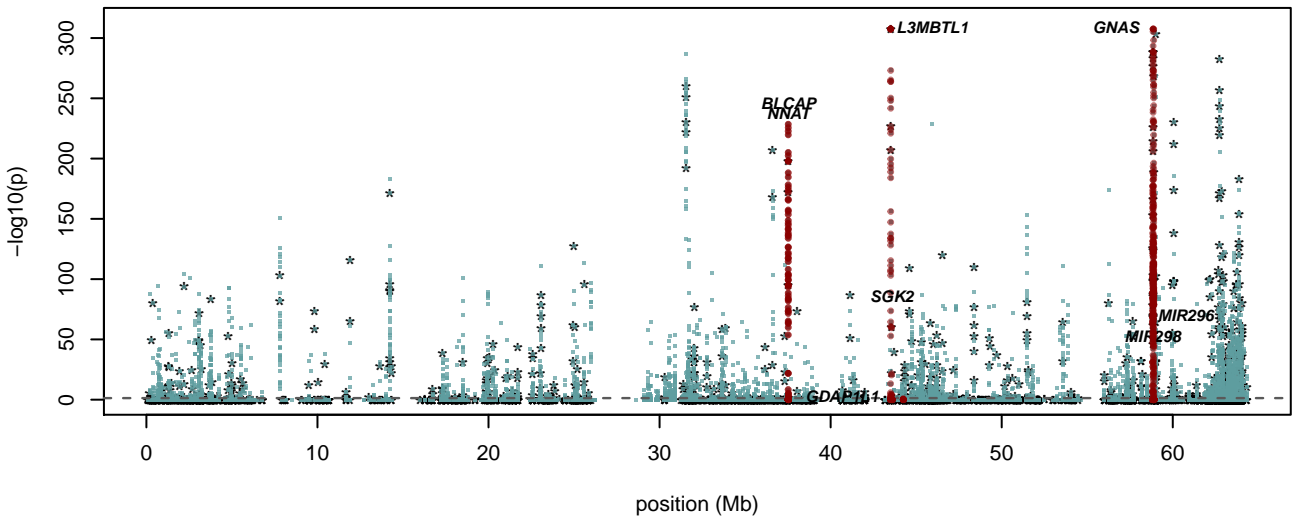

chr21

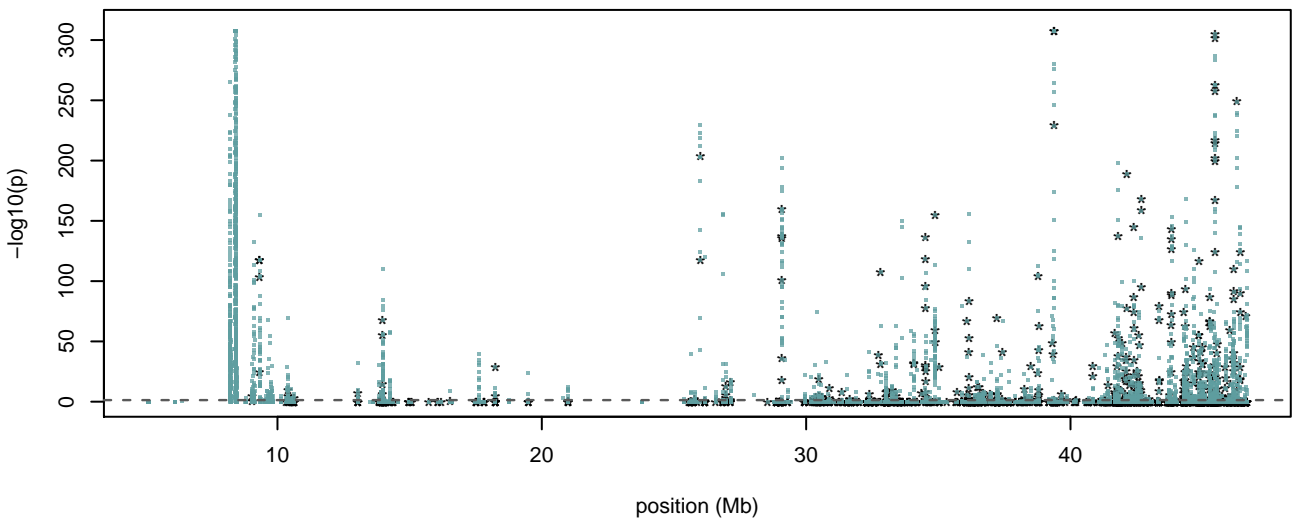

chr22

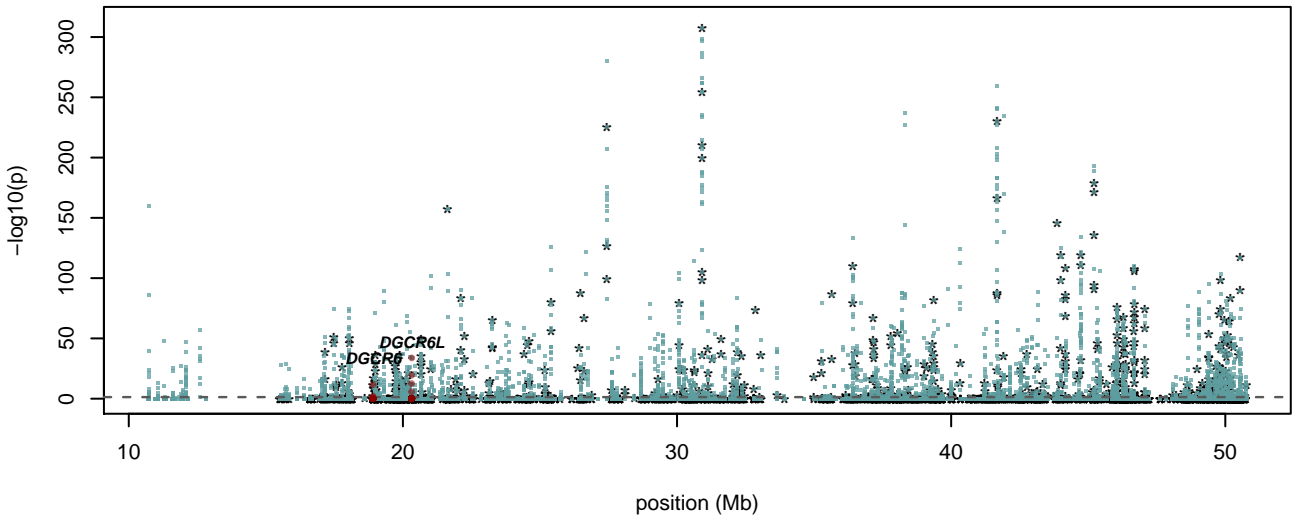

chrX

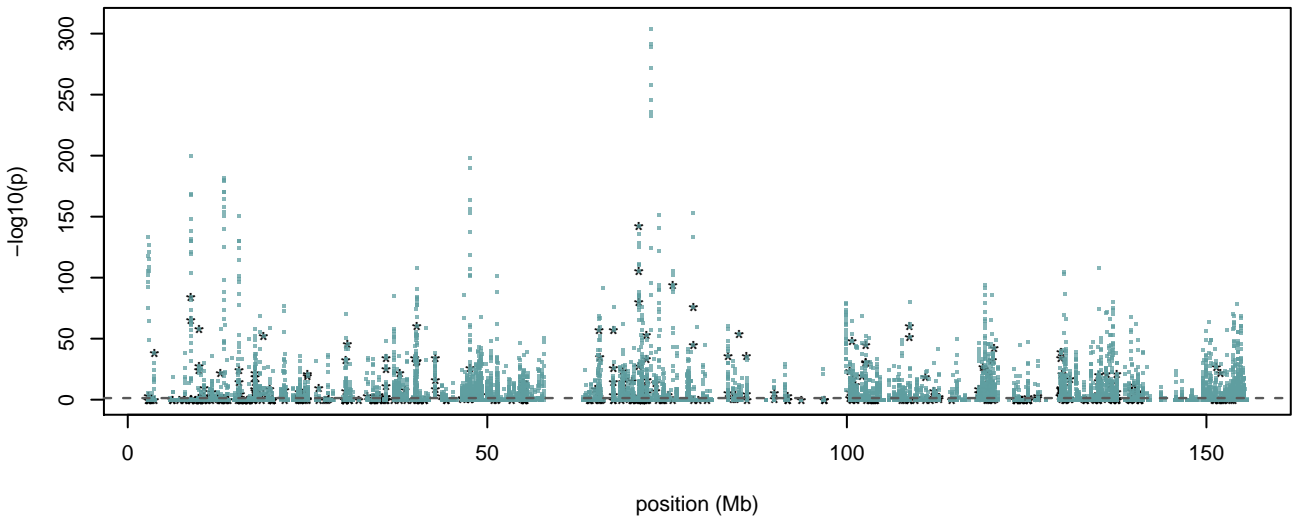

Supplement: Supplementary file 2 — Additional file 2: Figure S2. ‘Manhattan’ plots of ASM across each Chromosome. [file 13072_2019_304_MOESM2_ESM.pdf]
